# Supplementary material for: Development and Characterization of Inhaled Ethanol as a Novel Pharmacological Strategy Currently Evaluated in a Phase II Clinical Trial for Early-Stage SARS-CoV-2 Infection
Source: Pharmaceutics. 2021 Mar 5;13(3):342. doi: 10.3390/pharmaceutics13030342 (PMC7999202; doi:10.3390/pharmaceutics13030342)
Supplement: Supplementary file 1 [file pharmaceutics-13-00342-s001.pdf]

pharmaceutics

Supplementary Material: Development and Characterization of Inhaled Ethanol as a Novel Pharmacological Strategy Currently Evaluated in a Phase II Clinical Trial for Early-Stage SARS-CoV-2 Infection

Ana Castro-Balado, Cristina Mondelo-García, Letricia Barbosa-Pereira, Iria Varela-Rey, Ignacio Novo-Veleiro, Néstor Vázquez-Agra, José Ramón Antúnez-López, Enrique José Bandín-Vilar, Raquel Sendón-García, Manuel Busto-Iglesias, Ana Rodríguez-Bernaldo de Quirós, Laura García-Quintanilla, Miguel González-Barcia, Irene Zarra-Ferro, Francisco J. Otero-Espinar, David Rey-Bretal, José Ramón Lago-Quinteiro, Luis Valdés-Cuadrado, Carlos Rábade-Castedo, María Carmen del Río-Garma, Carlos Crespo-Diz, Olga Delgado-Sánchez, Pablo Aguiar, Gema Barbeito-Castiñeiras, María Luisa Pérez del Molino-Bernal, Rocío Trastoy-Pena, Rossana Passannante, Jordi Llop, Antonio Pose-Reino and Anxo Fernández-Ferreiro

Supplementary Material S1. Preliminary Clinical Studies

Methods: A group of 6 healthy volunteers (five men and one woman), with an age of 39 ± 16 years (24-63) and a BMI of 22 ± 2 kg/m², were divided in two groups and subjected to the administration of 15 minutes of oxygen therapy (3 volunteers 2L/min flow and 3 volunteers 3L/min flow through a face mask Ventimask® (Flexicare, Mountain Ash, United Kingdom), using ethanol 65° solution as humectant. Ethanol was vaporized by passing oxygen through the disposable humidifier, which, instead of water, contains the ethanol solution (Figure S1). Safety has been assessed by observing volunteers during the whole process and recording oxygen saturation and heart rate during inhalation time. These measurements were performed using a PM-100 Medisana® (Medisana, Barcelona, Spain) pulse oximeter. Otherwise, systolic and diastolic blood pressure have been recorded at the beginning and end of inhalation. In order to evaluate the ethanol passage to systemic route, a plasma determination was performed at the end of inhalation (Siemens, Flex® ETOH reagent cartridge; Siemens Healthcare Diagnostics, Madrid, Spain). Finally, volunteers were kept under observation during 30 min and were asked about tolerance and subjective perception during and after the monitored period.

Citation: Castro-Balado, A.; Mondelo-García, C.; Barbosa-Pereira, L.; Varela-Rey, I.; Novo-Veleiro, I.; Vázquez-Agra, N.; Antúnez-López, J.R.; Bandín-Vilar, E.J.; Sendón-García, R.; Busto-Iglesias, M.; et al. Development and Characterization of Inhaled Ethanol as a Novel Pharmacological Strategy Currently Evaluated in a Phase II Clinical Trial for Early-Stage SARS-CoV-2 Infection. *Pharmaceutics* **2021**, *13*

Publisher's Note: MDPI stays neutral with regard to jurisdictional claims in published maps and institutional affiliations.

Copyright: © 2021 by the authors. Submitted for possible open access publication under the terms and conditions of the Creative Commons Attribution (CC BY) license (http://creativecommons.org/licenses/by/4.0/).

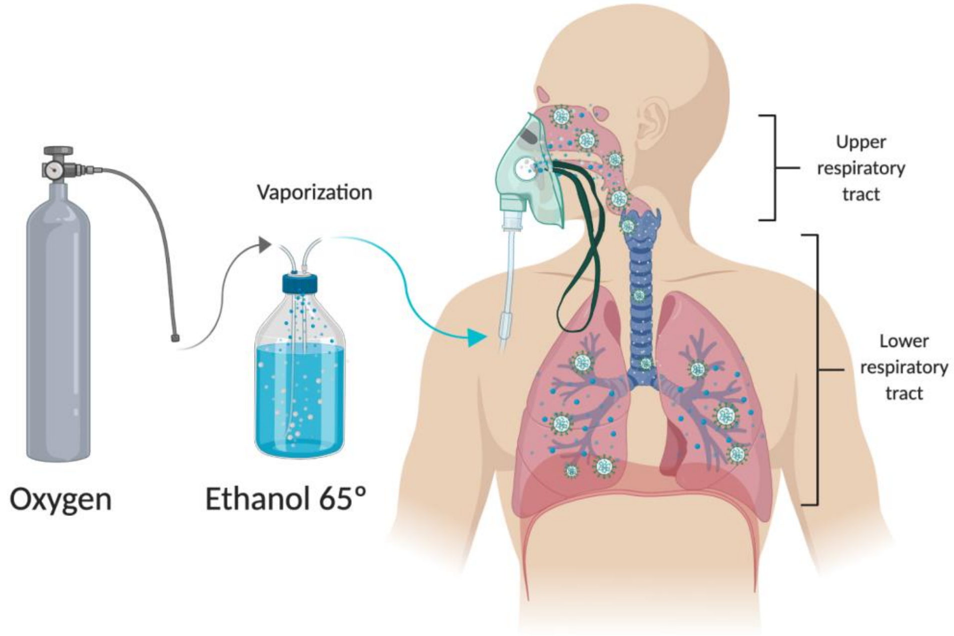

Figure S1. Therapeutic approach scheme to the use of inhaled ethanol. Created with BioRender.com.

Results: The inhalation of the hydro-alcohol mixture has been safe in both groups with no detected alterations of the physiological parameters. The heart rate was not altered during the inhalation period, with similar behaviors in both groups. On the other hand, the oxygen saturation rises slightly from the beginning of inhalation, something normal for being administering oxygen therapy. Neither systolic nor diastolic pressure undergo significant variations at the beginning and end after 15 minutes of inhalation, although a slight decrease is observed, more notable in the case of the group that receives ethanol at a flow of 3 L/min (Figure S2). Finally, no plasmatic concentrations of ethanol have been detected after the inhalation period. During inhalation-time subjects have not shown any psychomotor disturbance or any other effect. The subjective perception of the subjects has been positive in all cases without showing adverse reactions. The good results obtained on clinical safety must be interpreted with caution, since it involves a limited number of patients.

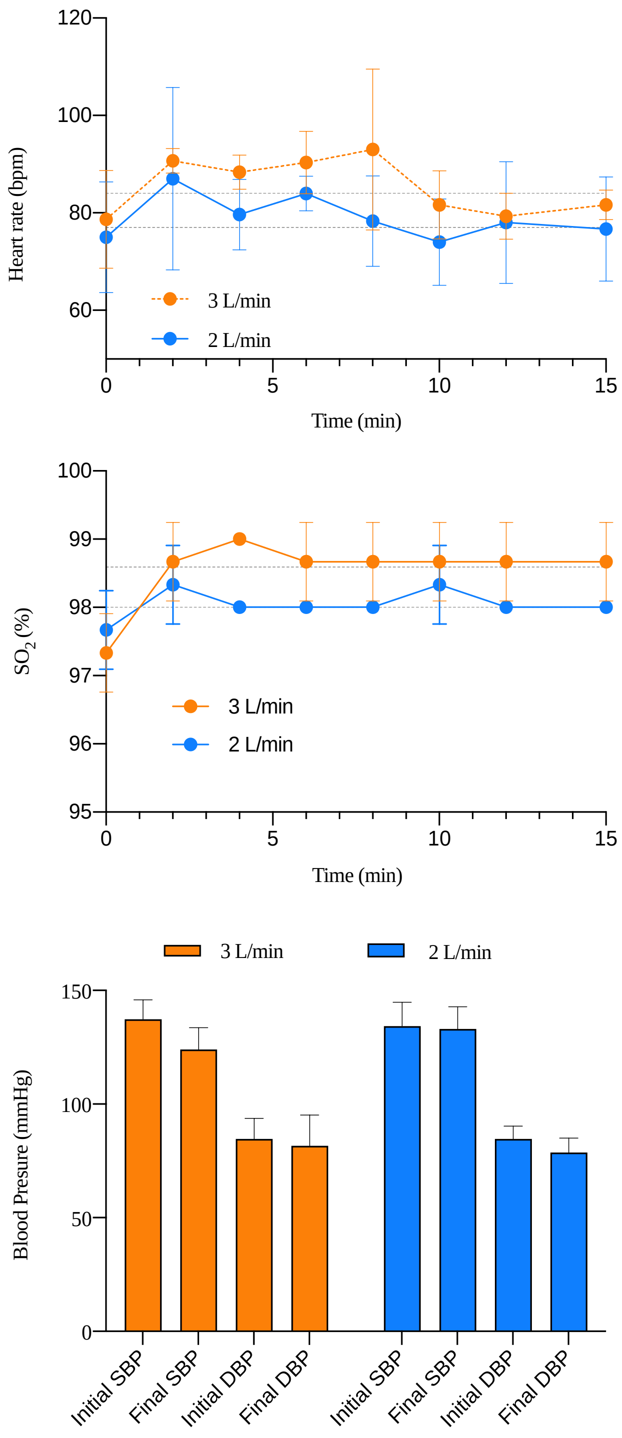

Figure S2. Physiological safety parameters monitored during inhalation. A. Heart Rate B. Oxygen Saturation C. Systolic Blood Pressure (SBP) and Diastolic Blood Pressure (DBP) before and after inhalation.
